# Supplementary material for: IPT9, a cis-zeatin cytokinin biosynthesis gene, promotes root growth
Source: Front Plant Sci. 2022 Oct 14;13:932008. doi: 10.3389/fpls.2022.932008 (PMC9616112; doi:10.3389/fpls.2022.932008)
Supplement: Supplementary file 1 [file Table_1.pdf]

Table S1. Primer sets used in this work

Purpose	Primer name	Primer sequence (5' → 3')	
		Forward primer (F)	Reverse primer (R)
Genotyping	KG7770	CTAAGCGACTCAATGGCGAAATCATCAG	CAGCGATAACTTCTGGAGAAGGTTTAG
	KG7770_LB	GTAATTCCCTTAATTCTCCGCTCATGATC	
	GK_302_F10	TTGTTTTGGTTTTGGTTTTGG	AGCTGTTAATTTGGCAGCATG
	LBpAC161	ATATTGACCATCATACTCATTGC	
Cloning	attB_IPT9	GGGGACAAGTTTGTACAAAAAAGCAGGCTA TGGTGATTGGCAGTGGCGT	GGGGACCACTTTGTACAAGAAAGCTG GGTTGCTATTGCGCTTTCCACGCA
	pDONR207	TCGCGTTAACGCTAGCATGGATCT	TAACATCAGAGATTTTGAGACAC
	pMDC32	TCATTTCATTTGGAGAGGACCT	TAACATAGATGACACCGCGC
	IPT9_cds		CTGTCTGTACCCAATGGCACGA
qPCR	oMP080	GGATTGTATCTGCGATGGTTTATGT	
	oMP081		CATGGGCCTCAGCGATAACT
