# Supplementary material for: IPT9, a cis-zeatin cytokinin biosynthesis gene, promotes root growth
Source: Front Plant Sci. 2022 Oct 14;13:932008. doi: 10.3389/fpls.2022.932008 (PMC9616112; doi:10.3389/fpls.2022.932008)
Supplement: Supplementary file 2 [file Table_2.pdf]

Supplemental Table 2. Multiple statistical comparisons between genotypes shown in Figure 3

Day 4				
Scion/ rootstock	Col-0 / Col-0	ipt2-9 / ipt29	ipt29 / Col-0	Col-0 / ipt29
Col-0 / Col-0		**	ns	**
ipt2-9 / ipt29			****	****
ipt29 / Col-0				***
Col-0 / ipt29				

Day 9				
Scion/ rootstock	Col-0 / Col-0	ipt2-9 / ipt29	ipt29 / Col-0	Col-0 / ipt29
Col-0 / Col-0		****	*	****
ipt2-9 / ipt29			****	****
ipt29 / Col-0				****
Col-0 / ipt29				

Day 5				
Scion/ rootstock	Col-0 / Col-0	ipt2-9 / ipt29	ipt29 / Col-0	Col-0 / ipt29
Col-0 / Col-0		**	ns	**
ipt2-9 / ipt29			****	**
ipt29 / Col-0				****
Col-0 / ipt29				

Day 10				
Scion/ rootstock	Col-0 / Col-0	ipt2-9 / ipt29	ipt29 / Col-0	Col-0 / ipt29
Col-0 / Col-0		***	**	****
ipt2-9 / ipt29			****	ns
ipt29 / Col-0				****
Col-0 / ipt29				

Day 6				
Scion/ rootstock	Col-0 / Col-0	ipt2-9 / ipt29	ipt29 / Col-0	Col-0 / ipt29
Col-0 / Col-0		**	ns	**
ipt2-9 / ipt29			****	*
ipt29 / Col-0				****
Col-0 / ipt29				

Day 11				
Scion/ rootstock	Col-0 / Col-0	ipt2-9 / ipt29	ipt29 / Col-0	Col-0 / ipt29
Col-0 / Col-0		***	*	****
ipt2-9 / ipt29			****	ns
ipt29 / Col-0				****
Col-0 / ipt29				

Day 7				
Scion/ rootstock	Col-0 / Col-0	ipt2-9 / ipt29	ipt29 / Col-0	Col-0 / ipt29
Col-0 / Col-0		****	**	****
ipt2-9 / ipt29			****	****
ipt29 / Col-0				****
Col-0 / ipt29				

Day 12				
Scion/ rootstock	Col-0 / Col-0	ipt2-9 / ipt29	ipt29 / Col-0	Col-0 / ipt29
Col-0 / Col-0		****	***	****
ipt2-9 / ipt29			****	ns
ipt29 / Col-0				****
Col-0 / ipt29				

Day 8				
Scion/ rootstock	Col-0 / Col-0	ipt2-9 / ipt29	ipt29 / Col-0	Col-0 / ipt29
Col-0 / Col-0		****	*	****
ipt2-9 / ipt29			****	****
ipt29 / Col-0				****
Col-0 / ipt29				

Day 13				
Scion/ rootstock	Col-0 / Col-0	ipt2-9 / ipt29	ipt29 / Col-0	Col-0 / ipt29
Col-0 / Col-0		****	ns	****
ipt2-9 / ipt29			****	*
ipt29 / Col-0				****
Col-0 / ipt29				

Asterisks indicate values significantly different in a Student's *t* test (*$p < 0.05$, **$p < 0.01$, ***$p < 0.001$, ****$p < 0.0001$).
