# Supplementary material for: IPT9, a cis-zeatin cytokinin biosynthesis gene, promotes root growth
Source: Front Plant Sci. 2022 Oct 14;13:932008. doi: 10.3389/fpls.2022.932008 (PMC9616112; doi:10.3389/fpls.2022.932008)
Supplement: Supplementary file 3 [file Presentation_1.pdf]

IPT9, a cis-Zeatin cytokinin biosynthesis gene, promotes root growth

Ioanna Antoniadi, Eduardo Mateo-Bonmatí, Markéta Pernisová, Federica Brunoni, Mariana Antoniadi, Mauricio Garcia-Atance Villalong, Anita Ament, Michal Karády, Colin Turnbull, Aleš Pěnčík, Karel Doležal, Karin Ljung, Ondřej Novák

Supporting information

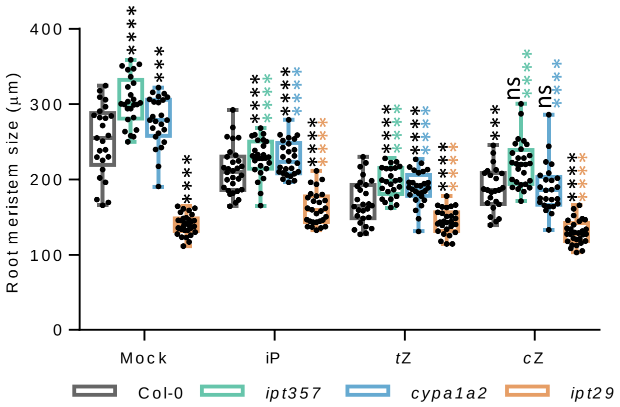

Supplementary Figure 1. Inhibitory effects of cytokinins on root growth are mimicked by root meristem size. Root meristem size of the wild-type Col-0, and the *ipt357*, *cypa1a2*, and *ipt29* multiple mutants grown for 7 days in media supplemented with 100 nM of iP, *tZ* and *cZ*. Black asterisks indicate values significantly different from Col-0 mock treatment and colour asterisks indicate significant differences from the corresponding mock genotype in a One-way ANOVA test (***p<0.001, ****p<0.0001; n ≥ 13).

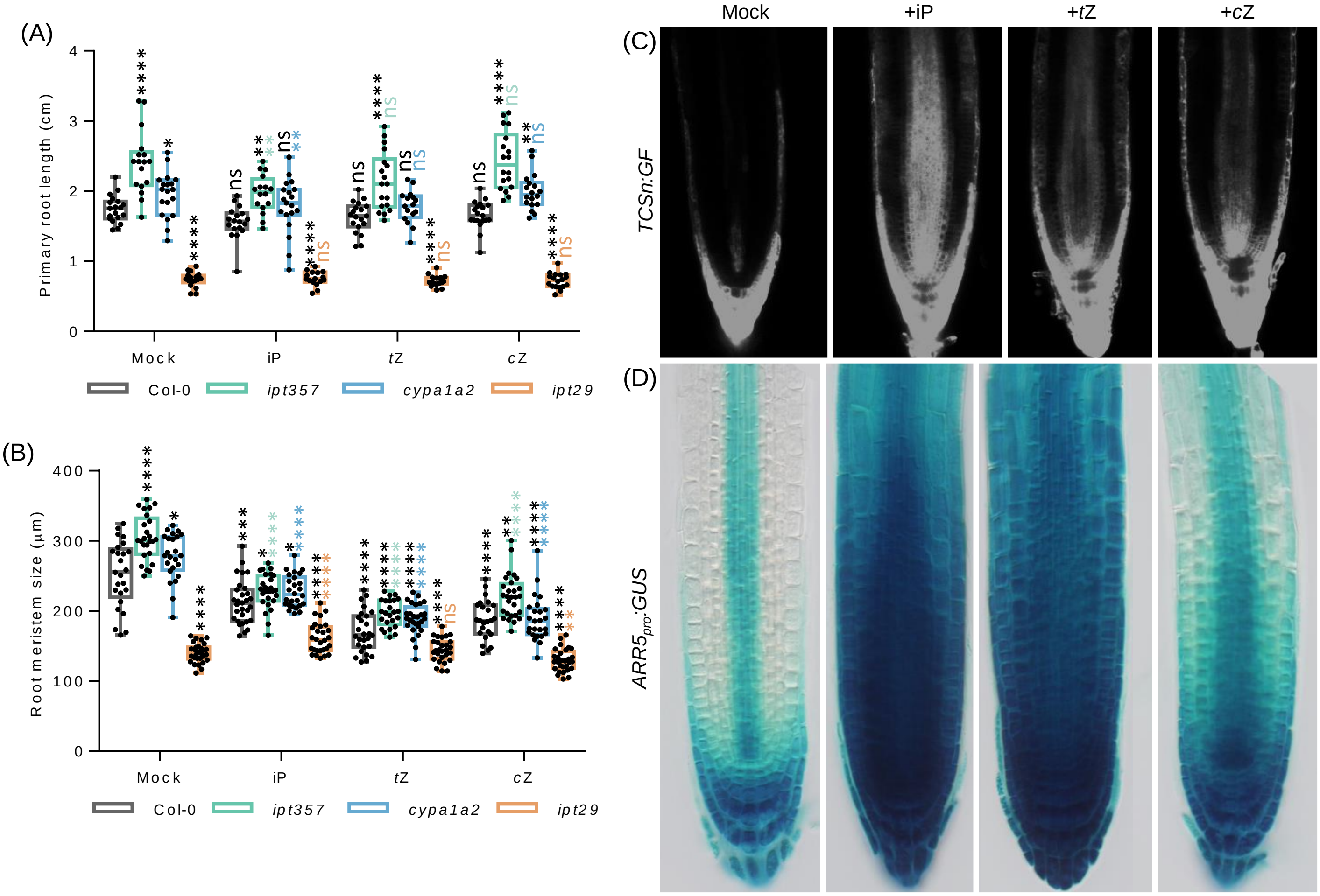

Supplementary Figure 2. 24 h treatment with different CKs inhibits root growth and triggers CK signalling. (A, B) Primary root length (A) and root meristem (B) of the wild-type Col-0, and the *ipt357*, *cypa1a2*, and *ipt29* multiple mutants grown for 6 days in standard MS media and 24 h in MS supplemented with 100 nM of iP, *tZ* and *cZ*. Black asterisks indicate values significantly different from Col-0 mock treatment and colour asterisks indicate significantly differences from the corresponding mock genotype in a One-way ANOVA test (*p<0.05, **p<0.01, ***p<0.001, ****p<0.0001; n ≥ 16). (C, D) Cytokinin signalling reporters *TCSn:GFP* (C) and *ARR5$_{pro}$:GUS* signal (D) after 24 h of treatment with different cytokinin types.

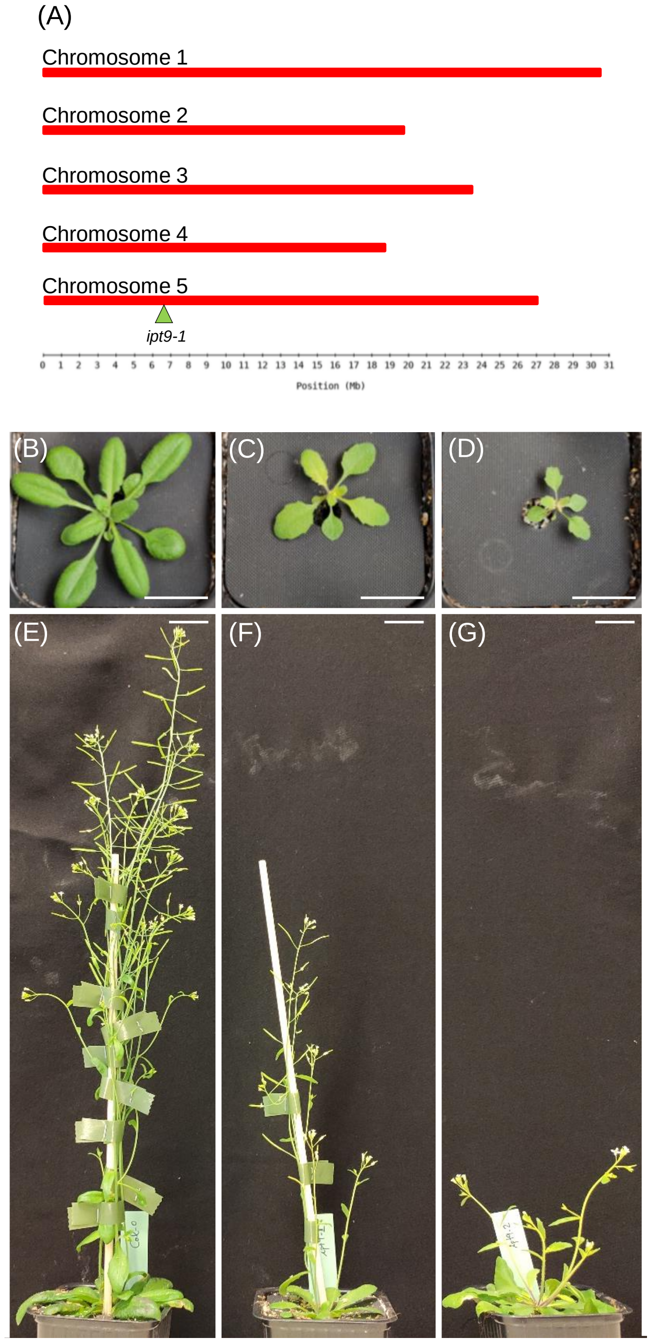

Supplementary Figure 3. Insertion number analysis performed in *ipt9-1* and shoot phenotypes of *ipt9* mutants. (A) Map of the five Arabidopsis chromosomes with indication of the position of the insertion found in *ipt9-1* (green triangle) using a tagged-sequence mapping strategy. (B-G) Shoot phenotype of the (B, E) wild-type Col-0 and the mutant (C, F) *ipt9-1*, and (D, G) *ipt9-2*. (L) Pictures were taken at (B-D) 23, and (E-G) 42 days. Scale bars indicate (B-D) 1 cm, and (E-G) 2 cm.

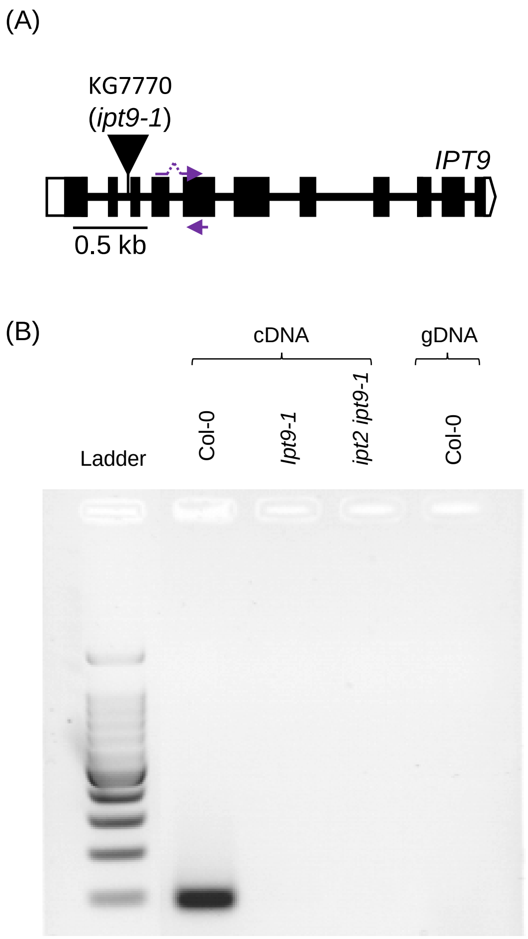

Supplementary Figure 4. **The *ipt9-1* is a knock-out mutant**. (A) Map of the *IPT9* gene with indication of the position of the *ipt9-1* insertion (black triangle) and the primers (not drawn to scale, in purple) used for semi-quantitative PCR (semi-qPCR). The forward primer is split between two exons to avoid amplification from genomic template. (B) Agarose gel showing the semi-qPCR products obtained after amplification with cDNA from Col-0, *ipt9-1*, and *ipt2 ipt9-1* and genomic Col-0.

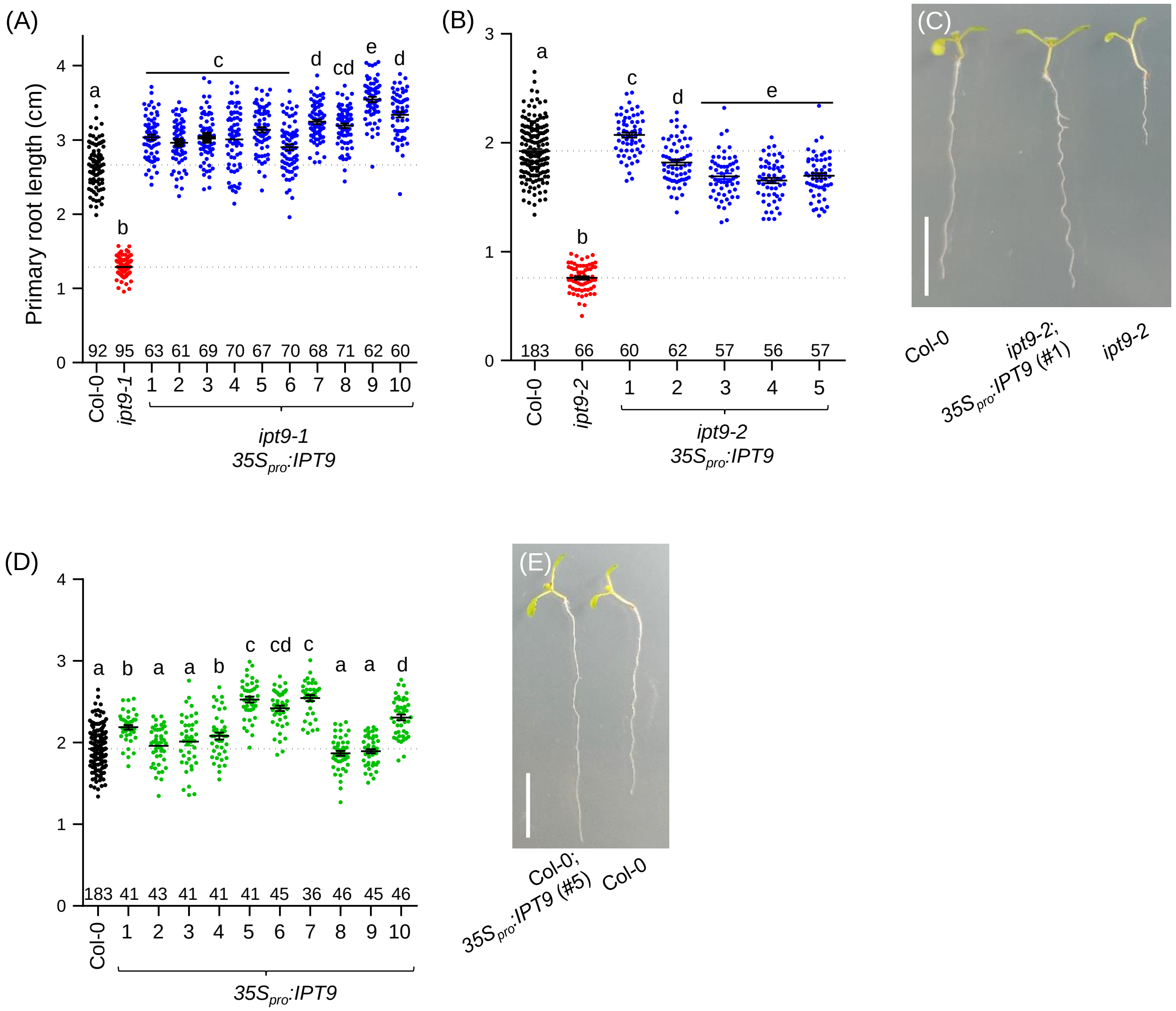

Supplementary Figure 5. Root phenotypic effects of the *35Spro:IPT9* transgene in mutant and wild-type backgrounds. (A, B, D) Primary root length quantification of independent transgenic families in the 7-day-old (A) *ipt9-1*, (B) *ipt9-2*, and (D) wild-type Col-0 background expressing *IPT9* under the constitutive 35S promoter. Small case letters indicate values significantly different ($p < 0.05$; n of each population is indicated above the genotype) in a Tukey's post-hoc test. (C, E) Primary root phenotype of transgenic *35S$_{pro}$:IPT9* in the (C) *ipt9-2*, and (E) Col-0 background. (F-K) Shoot phenotype of the (F, I) wild-type Col-0 and the mutant (G, J) *ipt9-1*, and (H, K) *ipt9-2*. (L) Map of the five Arabidopsis chromosomes with indication of the position of the insertion found in *ipt9-1* (green triangle) using a tagged-sequence mapping strategy. Scale bars indicate (C, E) 1 cm.
